# Neural Basis of Enhanced Executive Function in Older Video Game Players: An fMRI Study

**DOI:** 10.3389/fnagi.2017.00382

**Published:** 2017-11-21

**Authors:** Ping Wang, Xing-Ting Zhu, Zhigang Qi, Silin Huang, Hui-Jie Li

**Affiliations:** ^1^CAS Key Laboratory of Behavioral Science, Institute of Psychology, Beijing, China; ^2^Department of Psychology, University of Chinese Academy of Sciences, Beijing, China; ^3^Department of Radiology, Xuanwu Hospital, Capital Medical University, Beijing, China; ^4^Beijing Key Laboratory of Magnetic Resonance Imaging and Brain Informatics, Beijing, China; ^5^Institute of Developmental Psychology, Faculty of Psychology, Beijing Normal University, Beijing, China

**Keywords:** video game experience, older video game players, older non-video game players, fMRI, executive function

## Abstract

Video games have been found to have positive influences on executive function in older adults; however, the underlying neural basis of the benefits from video games has been unclear. Adopting a task-based functional magnetic resonance imaging (fMRI) study targeted at the flanker task, the present study aims to explore the neural basis of the improved executive function in older adults with video game experiences. Twenty video game players (VGPs) and twenty non-video game players (NVGPs) of 60 years of age or older participated in the present study, and there are no significant differences in age (*t* = 0.62, *p* = 0.536), gender ratio (*t* = 1.29, *p* = 0.206) and years of education (*t* = 1.92, *p* = 0.062) between VGPs and NVGPs. The results show that older VGPs present significantly better behavioral performance than NVGPs. Older VGPs activate greater than NVGPs in brain regions, mainly in frontal-parietal areas, including the right dorsolateral prefrontal cortex, the left supramarginal gyrus, the right angular gyrus, the right precuneus and the left paracentral lobule. The present study reveals that video game experiences may have positive influences on older adults in behavioral performance and the underlying brain activation. These results imply the potential role that video games can play as an effective tool to improve cognitive ability in older adults.

## Introduction

By the end of 2014, the number of individuals in the population over 60 years of age had already reached 212.42 million in China, accounting for 15.5% of the total population (National Bureau of Statistics of the People’s Republic of China, 2015). It is predicted that by the middle of this century, the older population will account for 35% of the total population in China (Wu, 2013). With the increasing number of older adults, age-related cognitive decline has been gaining increasing attention. Among the declining abilities, executive function seems to have the most ubiquitous consequences (Burgess et al., 1998), given that it is relevant for numerous functions such as planning, working memory, and mental flexibility as well as the monitoring of action (Chan et al., 2008) and adaptation to new situations (Collette et al., 2006). Braver and Barch (2002) claim that executive function may be responsible for age-related declines of the fluid abilities.

As a flexible and integrated training paradigm (Basak et al., 2008), video games, referring to games that use an audio-visual apparatus and that are based on a story (Esposito, 2005), have proven to be an effective tool in improving various cognitive abilities of older adults, including reaction time (RT), attention, visuospatial, memory, mental flexibility and global cognitive function (Dustman et al., 1992; Goldstein et al., 1997; Peretz et al., 2011; Kueider et al., 2012; Lampit et al., 2014; McDermott, 2014). Previous studies have also shown that video game playing is beneficial in improving executive function. Basak et al. (2008) found that older adults who were trained to play the video game *Rise of Nation* for 23.5 h showed significant improvement in task-switching, the N-back task and Raven’s Advanced Progressive Matrices task. Maillot et al. (2012) reported that compared to older non-video game players (NVGPs), older video game players (VGPs) presented significant improvement in executive function tasks, including the Stroop Test, the Matrix Reasoning Test, the Digit-Symbol Substitution Test, the Trail Making Test Part B-Part A and the Number Letter Sets Test after playing the *Nintendo Wii* for 24 h. In recent meta-analyses, Lampit et al. (2014) and Wang et al. (2016) have proven the positive impacts of video game experience on executive function in older adults.

Some neuroimaging studies have investigated the neural basis underlying the positive effects of video game experience. Tanaka et al. (2013) revealed positive relationships between the larger gray matter volume in the right posterior parietal cortex and the visual working memory task performance of VGPs. Kühn et al. (2014a) found a significant gray matter volume increase in the right hippocampus, the right dorsolateral prefrontal cortex and the bilateral cerebellum of the participants in a training group who played the video game *Super Mario* for 2 months. Moreover, they also found increased cortical thickness in left dorsolateral prefrontal cortex and left frontal eye fields in VGPs in adolescents (Kühn et al., 2014b). Due to video game playing, Granek et al. (2010) found increased prefrontal cortex activity when planning for complex eye-hand coordination tasks and the changing of the cortical networks for processing complex visually guided reaching. Gleich et al. (2017) found video game training might lead to decreased activation in dorsolateral prefrontal cortex and hippocampus in reward and frustration. In resting-state functional magnetic resonance imaging (fMRI) analysis, the increased functional connectivity between the attentional and sensorimotor networks (Gong et al., 2015) and the enhanced integration between the salience network and the central executive network (Gong et al., 2016) of VGPs compared to NVGPs were also found by researchers. However, most of relevant neuroimaging studies were based on young adults; few studies have investigated the neural mechanisms of video game effects on older adults. Therefore the neural mechanisms of the effects on the executive function of older adults remain unclear.

The present study aims to explore the functional neural correlates of the effects of video game experience on the executive function of older adults. We use the adapted version of the flanker task to test executive function (Eriksen and Eriksen, 1974) and to examine the differences in brain activity between older VGPs and older NVGPs. Increasing evidence shows that executive function depends on distributed frontal-parietal brain regions (Osaka et al., 2004; Sauseng et al., 2005; Desco et al., 2011), and therefore, we hypothesize that compared to older NVGPs, older VGPs will perform better on the flanker task and activate more in frontal-parietal brain regions.

## Materials and Methods

### Participants

Potential participants were recruited by means of posters in the community and online advertisements. All participants were healthy older adults, received no less than 6 years of education, had normal or corrected-to normal vision and met the criteria for participating in an MRI study, including no diagnosis of serious physical diseases (e.g., cardiovascular disease, neurological disease, disability), no claustrophobia and no metallic implants in the body (e.g., metal tooth, cardiac pacemaker) that could interfere with or cause injury due to the magnetic field. Those who played video games for more than 2 h (more than 0.5 h per day and at least 4 days) per week in the previous 6 months were defined as VGPs (Bailey et al., 2010; Rupp et al., 2016). NVGPs were participants who have no experience playing video games.

Forty-four participants were recruited in the present study, including 22 VGPs and 22 NVGPs. Four participants were excluded due to excessive head motion (more than 3 mm in either directions) during the scanning. Ultimately, a total of 40 participants (20 VGPs, 20 NVGPs) were included for further analysis. The demographic information for the participants in each group is listed in Table 1. There were no significant differences between the VGPs and the NVGPs in age, gender ratio, years of education or scores on the Mini-Mental State Examination (MMSE; Folstein et al., 1975), the Center for Epidemiologic Studies Depression scale (CES-D; Radloff, 1977), the State-Trait Anxiety Inventory (STAT; Spielberger et al., 1970) or the Activities of Daily Living Scale (ADL; Katz et al., 1963). VGPs showed higher hobby hours than NVGPs, therefore, it was controlled as a covariate in the following analyses.

**Table 1:** Demographic information for participants in each group.

	VGP (*N* = 20)		NVGP (*N* = 20)		*t*	*p*
	*M*	*SD*	*M*	*SD*		
Age (years)	65.00	5.97	63.75	6.66	0.62	0.536
Gender ratio (female)	50%	0.51	30%	0.47	1.29	0.206
Education (years)	13.20	3.37	11.35	2.68	1.92	0.062
MMSE	28.55	1.64	28.25	1.45	0.61	0.543
CES-D	10.95	5.39	11.25	4.05	−0.20	0.843
S-AI	26.30	5.25	26.20	10.23	0.04	0.969
T-AI	28.35	6.10	29.55	10.18	−0.45	0.065
ADL	8.15	0.37	8.00	0.00	1.83	0.075
IADL	12.25	0.72	12.10	0.85	0.60	0.550
Hobby hours	317.90	221.55	86.45	117.54	4.17	0.000***

Note: N, sample number; MMSE, Mini-Mental State Examination; CES-D, Center for Epidemiologic Studies Depression scale; S-AI, State-Anxiety Inventory; T-AI, Trait-Anxiety Inventory; ADL, Activities of Daily Living Scale; IADL, Instrumental Activities of Daily Living Scale; Hobby hours, total hours spent on other hobbies except video games. ***p < 0.001.

The study was approved by the local ethics committee of the Institute of Psychology, Chinese Academy of Sciences (IPCAS). Written informed consent was obtained from all participants in accordance with the Declaration of Helsinki prior to the study.

### Image Acquisition

The experiment was conducted on a 3T MRI scanner (GE Discovery MR750) in the Magnetic Resonance Imaging Research Center of the IPCAS. Foam padding was used to support the head and neck to minimize head motion and reduce cumulative head drift during the scanning session.

Task fMRI images were acquired using gradient-echo echo-planar imaging (EPI) sequences with a top-down sequential order according to the following parameters: repetition time (TR) = 2000 ms, echo time (TE) = 35 ms, flip angle (FA) = 60°, voxel size = 3.75 × 3.75 × 4 mm, slice thickness = 3.5 mm, slice gap = 0.5 mm, slice number = 39 slices, and field of view (FOV) = 24 × 24 cm^2^. In addition, T1 images and resting fMRI images were also acquired from the participants but not included in the data analysis in the present study. T1 images were acquired interleaved from bottom to up according to the following parameters: TR = 6.896 ms, TE = 2.99 ms, inversion time (TI) = 450 ms, FA = 8°, voxel size = 1 × 1 × 1 mm, slice thickness = 1 mm, slice number = 1 slice, and FOV = 25.6 × 25.6 cm^2^.

### Flanker Task

**Figure 1:**
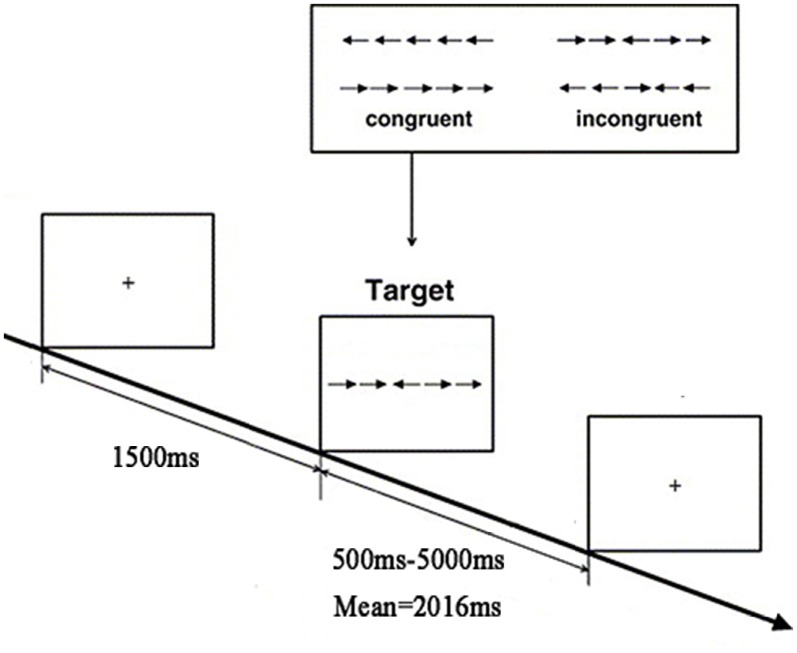
Flow chart of the functional magnetic resonance imaging (fMRI) task paradigm.

The experimental paradigm used in the present study was adapted from the Attention Network Test (ANT; Fan et al., 2005). We employed an event-related design in the paradigm, including a total of 240 trials for two runs, each of which lasted 7 min and 4 s. Details of the paradigm are illustrated in Figure 1. In each trial, a fixation cross was first presented for 1500 ms; then, the stimuli, consisting of a row of five arrowheads pointing either leftward or rightward, were presented varied from 500 ms to 5000 ms. The middle of the arrowhead pointed in either the same direction with or in different directions from the other four. The participants were required to identify the direction of the centrally presented arrow by pressing the number button “1” with the left index finger for the left direction or the number button “4” with the right index finger for the right direction. The trials could be classified into the congruent condition and the incongruent condition; the former indicates that the central arrowhead was flanked by two arrows on either side in the same direction, whereas the latter indicates that the central arrow and the flanker arrows were in different directions. The trials of these two conditions were presented randomly in an equal number of times in a run.

### Behavioral Data Analysis

The RT and accuracy of each task trial were recorded as the behavioral performance during the scanning. Following Fan et al. (2005), we used the difference of the average RT in the congruent condition subtracted from that in incongruent condition as the main measurement of executive function, and only the correct responses were included in the analyses. The between-group *t*-test was conducted using SPSS version 22.0 to examine the differences between the VGP and NVGP groups in behavioral performance. We also calculated the effect size (Cohen’s *d*, Cohen, 1988) of the behavioral task by comparing the standardized mean differences between VGPs and NVGPs. Based on Cohen’s *d*, an effect size of less than 0.2 was considered negligible, a value between 0.2 and 0.5 was considered small, a value between 0.5 and 0.8 was considered moderate, and a value equal to or greater than 0.8 was considered large (Cohen, 1988).

### fMRI Data Analyses

#### fMRI Data Pre-Processing

The fMRI data pre-processing was conducted with the SPM12 software package^1^. It included the following procedures: (1) Realignment. To correct the head motion of participants during the scan, rigid-body motion correction was conducted in three translational and three rotational directions. Any participants who moved more than 3 mm in any direction were excluded from further analysis. (2) Slice timing. With the middle slice, the 20th slice, as a reference slice, the acquisition timing difference was corrected for different locations. (3) Normalization. The EPI template is used for image normalization to eliminate the brain difference between individuals. (4) Smoothing. Spatial smoothing was applied with a Gaussian kernel of 8 mm full width at half maximum (FWHM) to remove high-frequency information, increase the signal-to-noise ratio for signal and reduce the mismatch across individuals.

#### Single-Subject Analysis

After pre-processing, the two-run image data of each subject were combined for further analysis. The first-level, single-subject analysis was conducted through linear regression on the 40 participants in the following three conditions, the congruent condition, the incongruent condition and the baseline condition (fixation cross), by specifying the onset time of each trial in the different conditions and using the six realignment parameters as covariates. After estimating the parameters, the design matrices of each subject in the three contrast conditions, the congruent-baseline, the incongruent-baseline and the congruent-incongruent, were ultimately obtained. We analyzed the brain activation in these three conditions, but given that the aim of the present study is to explore the neural substrates of executive function, we only reported the results in the incongruent-congruent condition.

#### Group Analysis

After the single-subject analysis, the group analysis was conducted on the between-group level. We first extracted the brain activation differences in the contrast of (incongruent condition − congruent condition) for each participant in the VGP group and the NVGP group. Then, the between-group *t*-test was conducted to identify the activation differences between VGPs and NVGPs; hobby hours were controlled as a covariate. The GRF correction with a voxelwise threshold of *p* < 0.01 (Qiu et al., 2016) was adopted using the DPABI software (Yan et al., 2016), and clusters were considered significant at a cluster threshold of *p* < 0.05.

### Correlation Analysis

In the correlation analysis, we considered the significant brain clusters in the group analysis to be regions of interest (ROI) and extracted the average beta values of these ROIs of each subject. To examine whether the activated brain regions were correlated with hobby hours and behavioral performance, we conducted the correlation analyses between each ROI and hobby hours as well as the flanker effects of all participants. To further examine whether the activated brain regions were influenced by total game hours, we conducted the correlation analyses between each ROI and the total game hours in VGPs.

### Functional Connectivity Analyses

In order to investigate the functional connectivity, we conducted the functional connectivity analyses between brain regions with significant group differences: left paracentral lobule, left lingual gyrus, left supramarginal gyrus, right dorsolateral prefrontal cortex, right precuneus, right angular gyrus and right inferior temporal gyrus. These regions were defined as ROIs. Furthermore, the left dorsolateral prefrontal cortex, left hippocampus, and right hippocampus were also taken as ROIs because these regions were reported to be related to video game experiences (Kühn et al., 2014a,b; Gong et al., 2015). We used the symmetrical coordinates of right dorsolateral prefrontal cortex as the coordinates of left dorsolateral prefrontal cortex, the left hippocampus (*x* = −22, *y* = −12, *z* = −20) and right hippocampus (*x* = 22, *y* = −12, *z* = −20) were defined according to previous study (Roy et al., 2009). These ROIs were defined as spheres (6-mm radius) around the local maxima within each region. We extracted the averaged BOLD time series separately from these ROIs in each participant, and then calculated the Pearson’s correlational coefficient between any two averaged time series of these ROIs. These correlational coefficients were transformed into Z-score with Fisher Z-transformation. We then compared the functional connectivity strength between VGPs and NVGPs with two-sample *t-test*.

### Gray Matter Volume Analyses

The gray matter volume analyses were calculated with SPM12. The structural images were first manually aligned to the AC-PC for maximizing registration accuracy. These realigned images were segmented into gray matter, white matter and CSF using default tissue probability maps provided with the software. The spatial normalization used linear and nonlinear Diffeomorphic Anatomical Registration Through Exponentiated Lie Algebra (DARTEL) registration. Gray matter partitions were finally smoothed with an 8-mm FWHM isotropic Gaussian kernel. The voxel-based comparisons were performed to identify the total brain gray matter volume between VGPs and NVGPs. We then compared the gray matter volume in brain regions with significant group differences in gray matter volume by using a two-sample two-tailed *t*-test. The same GRF correction with a voxelwise threshold of *p* < 0.01 and a cluster threshold of* p* < 0.05 was used.

## Results

### Behavioral Analyses

The behavioral analyses showed that the mean RT in the incongruent-congruent condition of the NVGPs (mean = 51.89 ms, SD = 32.90) was slower than that of the VGPs (mean = 31.16 ms, SD = 27.49). The independent-sample *t*-test suggested that the between-group difference was significant (*t* = −2.16, *p* = 0.037). The effect size was moderate (*d* = 0.68), indicating that VGPs presented moderate better than NVGPs.

### fMRI Analyses

**Figure 2:**
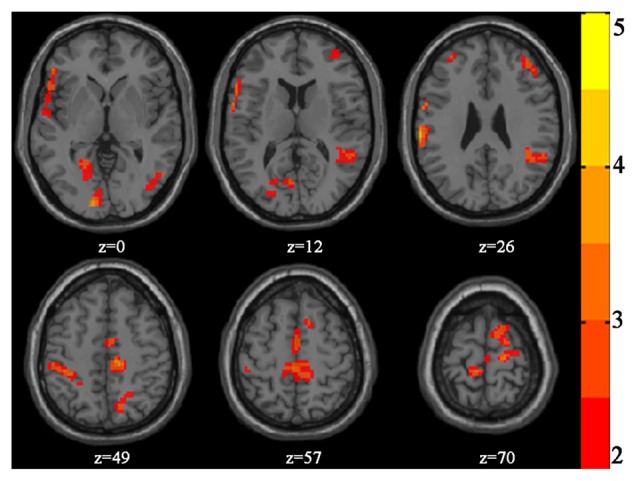
Increased activation of video game players (VGPs) in comparison with non-video game players (NVGPs) under incongruent-congruent condition.

Table 2 and Figure 2 present the significant clusters in which VGPs activated more than NVGPs in the incongruent-congruent condition in the fMRI analysis. As expected, the right dorsolateral prefrontal cortex, the left supramarginal gyrus, the right angular gyrus and the right precuneus in the parietal lobe and the left paracentral lobule crossing the frontal-parietal area were more greatly activated in VGPs than in NVGPs. Furthermore, VGPs also activated more in the right inferior temporal gyrus and the left lingual gyrus than NVGPs. No cluster activated more in NVGPs than in VGPs.

**Table 2:** Clusters VGPs activated stronger than NVGPs in incongruent-congruent condition.

Cluster	BA	Voxels	Peak MNI coordinates			Peak *T*-value
			*x*	*y*	*z*	
L paracentral lobule	4	698	0	−24	63	4.313
L lingual gyrus	18	610	−21	−72	−12	4.622
L supramarginal gyrus	48	589	−63	−24	33	5.004
R dorsolateral prefrontal cortex	45	348	36	54	21	4.067
R precuneus	7	328	21	−57	36	3.641
R angular gyrus	39	182	42	−42	30	3.926
R inferior temporal gyrus	37	140	51	−51	−6	3.510

* Note: L, left; R, right; BA, Brodmann area*.

### Correlation Analyses

**Figure 3:**
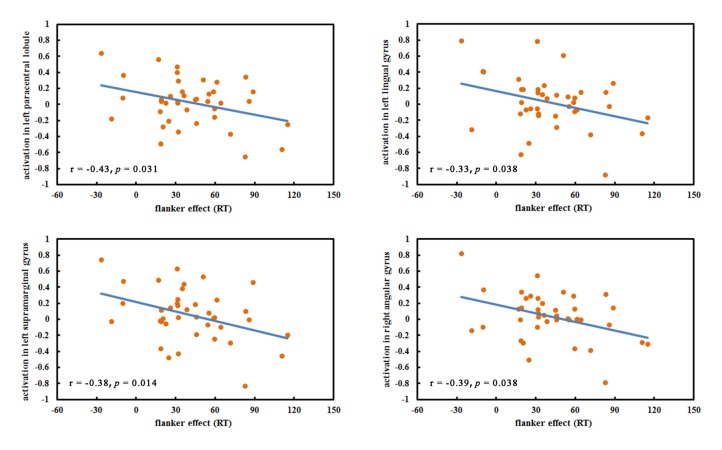
The significant correlations between regions of interests (ROIs) and flanker effect.

No significant correlations between hobby hours and the beta values in any ROI were found. The flanker effects were negatively correlated with the left paracentral lobule (*r* = −0.43, *p* = 0.031), the left lingual gyrus (*r* = −0.33, *p* = 0.038), the left supramarginal gyrus (*r* = −0.38, *p* = 0.014) and the right angular gyrus (*r* = −0.39, *p* = 0.014; Figure 3). Other ROIs were not significantly correlated with flanker effects. Furthermore, the correlations between the total game hours of VGPs and all ROIs were insignificant.

### Functional Connectivity Analyses

The results showed that VGPs showed increased functional connectivity between left paracentral lobule and right hippocampus (*r*_VGPs_ = 0.33, *r*_NVGPs_ = −0.04, *t* = 2.05, *p* = 0.047), left lingual gyrus and left supramarginal gyrus (*r*_VGPs_ = 0.62, *r*_NVGPs_ = 0.19, *t* = 2.03, *p* = 0.049) than NVGPs. Moreover, VGPs also showed decreased functional connectivity between right precuneus and right angular gyrus (*r*_VGPs_ = 0.35, *r*_NVGPs_ = 0.82, *t* = −2.20, *p* = 0.034) in comparison with NVGPs. No significant group differences were found in functional connectivity between other ROIs.

### Gray Matter Volume Analyses

No significant group differences were found between VGPs and NVGPs in total gray matter volume. We further performed ROI analyses. The aforementioned seven brain cluster (left paracentral lobule, left lingual gyrus, left supramarginal gyrus, right dorsolateral prefrontal cortex, right precuneus, right angular gyrus, right inferior temporal gyrus) existing group differences between VGPs and NVGPs during performing flanker task, together with left dorsolateral prefrontal cortex, left hippocampus and right hippocampus were taken as ROIs. No significant group differences were found in these ROIs.

## Discussion

In the present study, we examined the behavioral and brain activation differences between older VGPs and NVGPs when performing the flanker task. The results revealed that older VGPs showed relatively better behavioral performance and increased frontal-parietal activation compared to older NVGPs.

For the behavioral task performance, the flanker effect of NVGPs is significantly longer than that of VGPs; the effect size also shows moderate better behavioral performance in VGPs, confirming our hypothesis that video game experiences have a positive influence on executive function in older VGPs. This result replicates findings from the previous literature, proving the beneficial effects of video game playing on the executive function of older adults (Basak et al., 2008; Maillot et al., 2012). The current results also confirm our recent findings that older VGPs present outstanding performance on executive function tasks compared to older NVGPs (Wang et al., 2017).

As hypothesized, compared to NVGPs, VGPs activated more in the right dorsolateral prefrontal cortex. Previous studies have proven that the prefrontal cortex plays an important role in executive function (MacDonald et al., 2000; Fan et al., 2003; Nee et al., 2007). MacDonald et al. (2000) proposed that the dorsolateral prefrontal cortex is involved in resolving conflict. Fan et al. (2003) found that the left prefrontal cortex shows greater activation for the incongruent condition in the flanker task compared to the congruent condition. A neuroimaging meta-analysis aiming to investigate the underlying mechanism of executive function tasks, such as the flanker task, the go/no-go task and the Stroop task, revealed that the dorsolateral prefrontal cortex and the inferior frontal gyrus may be important regions that are responsible for the detection and/or resolution of interference (Nee et al., 2007). Zhu et al. (2010) compared brain activation between young and older adults when they performed the flanker task; similar to the present study, the results revealed that both the young and older adults activated the inferior frontal gyrus, but the extent was lower in the older adults. Compared to older NVGPs, given the stronger activation in the frontal area of VGPs found in the present study, we speculate that video game experience may improve the executive function of older adults and that the stronger activation in the right dorsolateral prefrontal cortex may contribute to the better performance.

VGPs were found to show increased activation in several parietal brain areas, including the left supramarginal gyrus, the right angular gyrus and the right precuneus. In a review on the neural substrates of executive function, Collette et al. (2006) stressed the importance of the parietal regions involved in executive function, in addition to the frontal areas. Salmon et al. (1996) found that the left supramarginal gyrus and the right angular gyrus were predominantly activated in the updating process and emphasized their significance in executive function. Moreover, both Ye and Zhou (2009) and Seghier (2013) concluded that the right angular gyrus was strongly activated in all conflict tasks. Therefore, we believe that the increased activation in parietal brain areas can help resolve conflict tasks and lead to better executive function performance.

Located in the frontal-parietal area, the paracentral lobule is the continuation of the precentral and postcentral gyri. Recent studies have shown that this area may be involved in executive function tasks. Zhang et al. (2015) reported the activation of the paracentral lobule in the stop signal task. Coderre and van Heuven (2013) found that the left paracentral lobule is sensitive to the Stroop effect and suggested that this area was involved in executive control. Therefore, we speculate that the stronger activation of the left paracentral lobule of older VGPs may also contribute to the better behavioral performance.

Gong et al. (2015) investigated the insula functional connectivity in young VGPs with resting-state fMRI data, they found young VGPs showed increased functional connectivity between insular subregions, and presented enhanced functional connectivity between attentional and sensorimotor networks. In the current study, we analyzed the functional connectivity with flanker task fMRI data and found older VGPs showed increased functional connectivity between left paracentral lobule and right hippocampus, left supramarginal gyrus and right dorsolateral prefrontal cortex. Moreover, we also observed decreased functional connectivity between right precuneus and angular gyrus in older VGPs. These results imply that the functional connectivity findings may also serve as the neural basis of behavioral performance improvement in addition to the brain activation induced by the flanker task. However, due to few studies investigated functional connectivity in older VGPs, the current results should be treated cautiously. Future studies are warranted to investigate the functional connectivity changes induced by the video game experiences.

No significant correlations between the ROIs and hobby hours across older VGPs and NVGPs were found, which excluded the potential influences of differences in hobby hours between VGPs and NVGPs on task performance and brain activation. The negative correlation between behavioral performance and the left paracentral lobule, the left lingual gyrus, the left supramarginal gyrus and the right angular gyrus suggested that the stronger activation of these regions was corresponding to the lower flanker effect and better executive function performance. The lack of correlations between the ROIs and total game hours suggested that longer video game experience did not necessarily contribute to the stronger activation in older VGPs. We speculate that there may exist an optimal number of game hours per week that leads to maximum benefits for executive function from video game experience and that extra video games playing will not continue to bring benefits for executive function. However, this speculation requires further investigation in the future.

Although there were no significant differences between older VGPs and NVGPs in age, gender, education and scores on the MMSE, CES-D, STAI and ADL, there are still some limitations in the present study. First, the sample size in each group was relatively small. Second, we only studied healthy older adults in the present study; whether our conclusion can extend to younger adults, persons with mild cognitive impairment or other clinical populations are unknown. Third, as a cross-sectional study, the present study can only reflect the positive relationships between video game experience and behavioral performance and brain activation during the flanker task. Future video game training studies are warranted to further determine the causal relationships between video game training and cognitive improvement and the underlying brain mechanisms.

## Conclusions

The present study revealed that older VGPs performed better in the flanker task and presented increased activation mainly in frontal-parietal regions compared to older NVGPs. The present results proved the positive impacts of video game playing on executive function in older adults, suggesting that video games may have the potential to play an effective role in improving executive function in older adults.

## Author Contributions

PW and H-JL conceived the idea and wrote the manuscript; PW and X-TZ collected the data; ZQ and SH contributed towards writing the article.

## Conflict of Interest Statement

The authors declare that the research was conducted in the absence of any commercial or financial relationships that could be construed as a potential conflict of interest.

## Footnotes

^1^http://www.fil.ion.ucl.ac.uk/spm/
